# Faecal Carriage of Gram-Negative Multidrug-Resistant Bacteria among Patients Hospitalized in Two Centres in Ulaanbaatar, Mongolia

**DOI:** 10.1371/journal.pone.0168146

**Published:** 2016-12-12

**Authors:** Bayaraa Baljin, Ganbaatar Baldan, Battogtokh Chimeddorj, Khosbayar Tulgaa, Batbaatar Gunchin, Tsogtsaikhan Sandag, Klaus Pfeffer, Colin R. MacKenzie, Andreas F. Wendel

**Affiliations:** 1 Department of Microbiology and Immunology, School of Biomedicine, Mongolian National University of Medical Sciences, Ulaanbaatar, Mongolia; 2 Department of Molecular Biology and Genetics, School of Biomedicine, Mongolian National University of Medical Sciences, Ulaanbaatar, Mongolia; 3 Institute of Medical Microbiology and Hospital Hygiene, University Hospital, Heinrich-Heine-University, Düsseldorf, Germany; Ross University School of Veterinary Medicine, SAINT KITTS AND NEVIS

## Abstract

Gram-negative multidrug-resistant organisms (GN-MDRO) producing β-lactamases (ESBL, plasmid-mediated AmpC β-lactamases and carbapenemases) are increasingly reported throughout Asia. The aim of this surveillance study was to determine the rate of bacterial colonization in patients from two hospitals in the Mongolian capital Ulaanbaatar. Rectal swabs were obtained from patients referred to the National Traumatology and Orthopaedics Research Centre (NTORC) or the Burn Treatment Centre (BTC) between July and September 2014, on admission and again after 14 days. Bacteria growing on selective chromogenic media (CHROMagar ESBL/KPC) were identified by MALDI-ToF MS. We performed susceptibility testing by disk diffusion and PCR (*bla*_IMP-1_, *bla*_VIM_, *bla*_GES_, *bla*_NDM_, *bla*_KPC_, *bla*_OXA-48_, *bla*_GIM-1_, *bla*_OXA-23_, *bla*_OXA-24/40_, *bla*_OXA-51_, *bla*_OXA-58_, *bla*_OXA-143_, *bla*_OXA-235_, *bla*_CTX-M_, *bla*_SHV_ *bla*_TEM_ and plasmid-mediated *bla*_AmpC_). Carbapenemase-producing isolates were additionally genotyped by PFGE and MLST. During the study period 985 patients in the NTORC and 65 patients in the BTC were screened on admission. The prevalence of GN-MDRO-carriage was 42.4% and 69.2% respectively (*p*<0.001). Due to the different medical specialities the two study populations differed significantly in age (*p<*0.029*)* and gender (*p*<0.001) with younger and more female patients in the burn centre (BTC). We did not observe a significant difference in colonization rate in the respective age groups in the total study population. In both centres most carriers were colonized with CTX-M-producing *E*. *coli*, followed by CTX-M-producing *K*. *pneumoniae* and CTX-M-producing *E*. *cloacae*. 158 patients from the NTORC were re-screened after 14 days of whom 99 had acquired a new GN-MDRO (*p*<0.001). Carbapenemases were detected in both centres in four OXA-58-producing *A*. *baumannii* isolates (ST642) and six VIM-2-producing *P*. *aeruginosa* isolates (ST235). This study shows a high overall prevalence of GN-MDRO in the study population and highlights the importance of routine surveillance, appropriate infection control practice and antibiotic prescribing policies to prevent further spread especially of carbapenemases.

## Introduction

Antimicrobial resistance is an increasing threat to health systems worldwide leading to treatment failure, high treatment costs and increased mortality. The rise of β-lactamase-producing Gram-negative multidrug-resistant organisms, such as extended-spectrum β-lactamase (ESBL)-, plasmid-mediated AmpC β-lactamases or carbapenemase-producing bacteria is of particular concern [1]. The main reservoirs of the different Gram-negative species are the environment and the gut of animals and humans and these organisms may cause health-care associated or community-acquired infections in humans. Due to the presence of co-resistance to other antibiotic classes (especially fluoroquinolones and aminoglycosides) the antibiotic treatment of infections caused by these organisms is a challenge [2]. The dissemination of these β-lactamase enzymes is facilitated by both horizontal transfer via mobile genetic elements (integrons, transposons and plasmids) and bacterial clonal proliferation [3]. Globally antimicrobial resistance surveillance data varies from country to country, ranging from no data to effective national surveillance programs [1]. Asia, which is known for its high prevalence of ESBL-producing Enterobacteriaceae, metallo-β-lactamases like NDM-1 and nosocomial multidrug-resistant *A*. *baumannii*, represents one of the epicentres of antimicrobial drug resistance [4]. The ESBL carriage rates in the community in Asia are amongst the highest worldwide (exceeding 50% in some studies), the lowest reported from Europe and North America [2, 4–6]. With regards to Mongolia, the monitoring system of multidrug-resistant bacteria is less well developed, mainly due to a limited diagnostic infrastructure. Cultures are only taken when empiric antibiotic therapy fails and numbers of hospital-associated infections are certainly underestimated [7, 8]. There is evidence pointing to presence of ESBL- and carbapenemase-producing bacteria in Mongolia. A previous study has shown the spread of multidrug-resistant *A*. *baumannii*, mainly carrying *bla*_OXA-23_-like or *bla*_OXA-58_-like genes, in hospitals [9]. Other studies have reported the presence of CTX-M enzymes in clinical *E*. *coli* and *Klebsiella* isolates in clinical samples [10, 11]. Additionally one of the major risk-factors for the development of resistance, the uncontrolled use and misuse of antimicrobial drugs, is widespread in Mongolia [12, 13].

The aim of this cross-sectional study is to determine the carriage rate of β-lactamase-producing Gram-negative multidrug-resistant bacteria (GN-MDRO) by patients on hospital admission and the frequency of nosocomial acquisition. Subsequently the main mechanisms of β-lactamase drug-resistance were explored among these GN-MDRO. In this study the term GN-MDRO is defined as non-susceptibility to at least three antimicrobial categories as defined by Magiorakis et al. [14].

## Materials and Methods

### Setting and sampling

The study was performed at two tertiary care hospitals providing specialized care located in Ulaanbaatar, between July and September 2014. The National Hospital of Traumatology and Orthopaedics (NTORC) has 260 beds and is divided into seven subunits/wards (intensive care unit, neurosurgery, spinal surgery, orthopaedics, hand surgery, paediatric surgery and trauma surgery). The 60-bed Skin Burn Treatment Centre (BTC) is comprised of three wards (children, adults and intensive care). Rectal swabs (eSwab, Copan Diagnostics, Italy) were taken from in-patients referred to both hospitals on admission (up to the 3^rd^ day of admission) and a second sample collection was done of those remaining in the hospital after two weeks (day 14).

### Strain identification and susceptibility testing

Swabs were cultured on selective chromogenic media (CHROMagar ESBL and KPC, CHROMagar, France) and plates were incubated for 24 h at 37°C. Bacteria with a distinct colonial morphology and/or colour on media were subcultured on blood agar and were further processed for identification (MALDI-TOF MS, and, in case of ambiguous results, VITEK 2 system, bioMérieux, Germany). Antibiotic susceptibility testing (AST) by disk diffusion method (Becton Dickinson, Germany) was performed on Mueller-Hinton agar with the antibiotics: cefotaxime, ceftazidime, cefoxitin, ertapenem, imipenem, meropenem, ciprofloxacin, gentamicin, tobramycin, tigecycline, chloramphenicol, nitrofurantoin and trimethoprim-sulfamethoxazole. In case of carbapenemase production we further performed a colistin Etest (bioMérieux, Marcy l’Etoile, Germany). EUCAST breakpoints were used for AST result interpretation. In Enterobacteriaceae β-lactamase resistance genes were identified following the EUCAST screening cut-off values for ESBL, AmpC β-lactamases and carbapenemases. Exceptions to this were Enterobacteriaceae with known inducible chromosomal AmpC β-lactamase (*Enterobacter* spp., *C*. *freundii*, *M*. *morganii*, *P*. *stuartii*, *Serratia* spp. or *H*. *alvei*), where EUCAST screening cut-off values for cefoxitin were not applied [15]. In *Pseudomonas* spp. molecular detection of carbapenemases was performed if the isolate was non-susceptible to ceftazidime and at least one carbapenem (imipenem and/or meropenem). In *Acinetobacter* spp. molecular detection of carbapenemases was performed if the isolate was non-susceptible to imipenem and/or meropenem.

### Molecular detection of β-lactamase genes

DNA preparation was performed from one colony by heating in a volume of 100 μl tris buffer at 95°C for 10 minutes followed by centrifugation at 12000 × *g*. Based on the AST results the supernatant was subjected to the following conventional and real-time multiplex PCRs to detect the ESBL-, AmpC β-lactamase- and carbapenemase-encoding genes: *bla*_IMP-1_, *bla*_VIM-1_-like, *bla*_VIM-2_-like, *bla*_GIM-1_, *bla*_NDM_, *bla*_GES_, *bla*_KPC_ and *bla*_OXA-48_ [16, 17]; *bla*_CTX-M-1_, *bla*_CTX-M-2_, *bla*_CTX-M-9_ and *bla*_CTX-M-8/-25_ groups [18]; different plasmid-mediated *ampC* gene subgroups (*bla*_ACC_, *bla*_FOX_, *bla*_CMY_, *bla*_DHA_, *bla*_MOX_, *bla*_LAT_, *bla*_BIL-1_, *bla*_ACT-1_ and *bla*_MIR-1_) [19]. If results were negative for *bla*_CTX-M_, PCRs for *bla*_TEM_ and *bla*_SHV_ [20] were performed. *Acinetobacter* spp. were additionally tested for relevant *bla*_OXA_ genes (*bla*_OXA-23_, *bla*_OXA-24/40,_ *bla*_OXA-51_, *bla*_OXA-58_, *bla*_OXA-143_ and *bla*_OXA-235_) as described previously [21, 22]. In case of the isolated detection of *bla*_OXA-51_ gene we further checked for IS*Aba1* upstream of and adjacent to *bla*_OXA-51_-like genes as previously described [23].

### Phenotypic detection of ESBL, AmpC β-lactamases and carbapenemases

In the case of inconclusive results (AST pointing to a certain resistance mechanisms, but negative PCR) confirmatory phenotypic methods were applied as follows: for ESBL a combined-disk test (CDT) proposed by EUCAST [15] or MIC test strip cefepime/cefepime + clavulanic acid (Liofilchem, Italy); for AmpC β-lactamases a MIC test strip cefotetan/cefotetan + cloxacillin (Liofilchem, Italy); for carbapenemases an MBL imipenem/imipenem + EDTA MIC test strip (Liofilchem, Italy); or a modified-Hodge-Test as recommended by CLSI with a 10 μg meropenem disk and/or a Rapidec^®^ CarpaNP Test (biomérieux, France).

### Genotyping of carbapenemase-producing bacteria

The genetic relatedness of the carbapenemase-producing isolates was investigated using MLST as described previously for *A*. *baumannii* [24] and *Pseudomonas aeruginosa* [25] Furthermore PFGE with *SpeI* was performed of the VIM-2 producing *P*. *aeruginosa* isolates. DNA separation was performed in 1% agarose in 0.5× TBE buffer using a CHEF-DR III System (Bio-Rad, La Jolla, CA, USA) as described previously [16] under the following conditions: 6 V/cm with an angle of 120° for 22 h with pulse times of 5 s to 45 s. As a marker we used a (45.5 kb– 1 Mb) Lambda ladder from Biolabs (New England Biolabs, Hitching, UK). The strain relatedness was calculated with GelCompar II software (version 5.1) in accordance with the Tenover criteria [26]. Epidemiologically non-related clinical strains were used as a reference.

### Genetic characterisation of VIM-2-producing *P*. *aeruginosa*

The complete class 1 integron sequence containing the *bla*_VIM-2_ cassette was resolved with primers targeting conserved-sequences and the *bla*_VIM-2_ gene cassette. At the 3’-end we not only targeted the common class 1 integron 3’-end composed of the *qacE*Δ*1* gene overlapping with the *sul1* gene, but also the *tniC* gene associated in rare cases with VIM-2-carrying integrons (e.g. described in [27]). The primers used are shown in Table 1. The PCR protocol included 5 min denaturation at 95°C followed by 35 cycles of 1 min 95°C denaturation, 1 min 60°C annealing and 1 min 72°C elongation. Sequencing was performed at the Biological and Medical Research Centre (BMFZ) core facility of the University of Düsseldorf using the Sanger technique with cycle sequencing and dye-marked terminators (BigDyes, Applied Biosystems, Darmstadt, Germany) using a 3130 xl Genetic Analyser (Applied Biosystems). Gel plugs of chromosomal DNA were prepared for the PFGE and S1-nuclease digestion as previously described and in-gel hybridisation was performed using a ^32^P-radiolabelled *bla*_VIM-2_-probe (product of primers VIM2S1-F1 and VIM2S1-R2 (Table 1)) [16].

**Table 1:** Primers used in this study for PCR and sequencing.

Primer	Sequence (5'→3')	Target	Possible Combinations	Source
IntI1-F	GCCGTAGAAGAACAGCAAGG	*intI1*	paired with VIM2-R	[28]
VIM2S1-F1	ACCAGATTGCCGATGGTGTT	*bla*_VIM-2_	paired with VIM2S1-R2	this study
VIM2-R	CTCGCAGACGGGACGTACA	*bla*_VIM-2_	paired with IntI1-F	[16]
VIM-883-F	GGAACGTGGCCGATGCCGAT	*bla*_VIM-2_	paired with 3'-end	this study
VIM2S1-R2	AGCGATTTGTGTGCGCTTTT	*bla*_VIM-2_	paired with VIM2S1-F1	this study
tniC-R4	ATGACCGCCGCCAAGCTACG	*tniC*	paired with VIM-883-F	this study
qacup	TGCATAAGCAACACCGACAG	*qacE*Δ*1*	paired with VIM-883-F	[28]
sulI1-R	GGCTCTCATCGAAGAAGGAG	*sul1*	paired with VIM-883-F	[16]

### Statistical analysis

Statistical analysis was performed with SPSS 22.0.0. The Student *t* test was used for continuous normally distributed (parametric) variables, the Mann-Whitney *U* test for variables that did not follow a normal distribution and the χ^2^ test or Fisher's exact test for categorical variables. The McNemar test was used to compare colonization rates at admission and after 14 days. All association tests were 2-tailed and a value of *p*<0.05 was considered to be statistically significant. 95% confidence intervals for proportions were calculated based on binomial distribution.

### Nucleotide sequence accession number

The *bla*_VIM-2_ integron sequence was submitted to GenBank and has been allocated the accession number KT768111.

### Ethic statement

The study was approved by the Ethics committee of the Mongolian National University of Medical Sciences. Written informed consent was obtained from each patient (if minor from the parents), before swabs were taken.

## Results

Percentages are rounded to one decimal place in the presentation of the results.

### Patient characteristics

During the study period, a total of 1050 patients were screened for GN-MDRO carriage. 985 patients were sampled in the trauma hospital (NTORC), 65 patients in the burn hospital (BTC) at admission. 158 out of 985 patients from the NTORC were re-screened after 14 days. In the BTC no second swabs were obtained. The basic patient characteristics and significance values are displayed in Table 2. The two hospital populations differed significantly in gender, age and sex.

**Table 2:** Patient characteristics of the study population with univariable analysis of the two study sites.

Characteristics of patients	Total (n = 1050)	NTORC (n = 985)	BTC (n = 65)	*p*-value
Female gender (%)	31.6	30.8	44.6	0.029
Age, years (mean, SD)	41, 16.6	42.2, 15.4	22, 22	<0.001
Age, years (median, min, max)	40, 0.5, 90	41, 2, 90	19, 0.5, 68	
GN-MDRO carriers (%)	463 (44.2)	418 (42.4)	45 (69.2)	<0.001
patiens with > 1 GN-MDRO (%)	77 (7.3)	56 (5.7)	21 (32.3)	<0.001

### Prevalence of MDRO at admission and after 14 days

The prevalence of carriage of GN-MDRO on admission was 42.4% (95% CI [39.3, 45.5]) in the NTORC and 69.2% (95% CI [58, 80.4]) in the BTC (Table 2). The overall prevalence was 44.2% (95% CI [41.2, 47.2]). Significantly more patients in the BTC compared to the NTORC carried more than one GN-MDRO (32.3% vs. 5.7% resp., *p*<0.001) (Table 2). Analysis of colonization in different age subgroups (<6 years, 7–17 years, 18–59 years and >60 years) did not show a significant difference in the total study population or the NTORC. In the BTC children (< 6 years) were significantly less colonized with GN-MDRO than adults (18–60 years) (51.7% vs. 82.8% resp.; *p* = 0.012).

Of the 158 patients from the NTORC from whom an additional sample was obtained at day 14, 110 were GN-MDRO-positive after 14 days. A significant number of patients (n = 99, *p*<0.001) acquired a new GN-MDRO, either a GN-MDRO for the first time or a second new strain. Additionally, on the 14^th^ hospital day the percentage of patients that carried more than one strain rose from 3.1% to 13.3% (*p* = 0.001).

### Characterization of species and β-lactamase genes

A total of 478 (NTORC) and 71 (BTC) different β-lactamase-producing GN-MDRO were collected on admission; on day 14 (NTORC) 133 different β-lactamase-producing GN-MDRO were collected. A detailed overview of the species/resistance mechanism combinations of all three groups are depicted in Table 3.

**Table 3:** GN-MDRO grouped by species and resistance mechanisms at the two sampling sites.

Species	β-lactamase	NTORC admission (n = 478) No. (%)	NTORC day 14 (n = 133) No. (%)	BTC admission (n = 71) No. (%)
**Enterobacteriaceae**				
*E*.*coli*	CTX-M-1 group	163 (34.1)	35 (26.3)	12 (16.9)
	CTX-M-2 group	2 (0.4)	2 (1.5)	-
	CTX-M-9 group	237 (49.6)	48 (36.1)	28 (39.4)
	CTX-M-8/-25 group	1 (0.2)	-	-
	CMY-2	2 (0.4)	-	-
	DHA-1	-	1 (0.8)	-
	CTX-M-1 group + CTX-M-9 group	5 (1.0)	1 (0.8)	-
	CTX-M-9 group + CMY-2	2 (0.4)	-	-
*K*. *pneumoniae*	CTX-M-1 group	12 (2.5)	8 (6.0)	7 (9.9)
	CTX-M-9 group	10 (2.1)	7 (5.3)	11 (15.5)
*K*. *oxytoca*	CTX-M-1 group	2 (0.4)	-	-
	CTX-M-2 group	1 (0.2)	-	-
	CTX-M-9 group	5 (1.0)	1 (0.8)	-
*E*. *cloacae*	CTX-M-1 group	11 (2.3)	10 (7.5)	2 (2.8)
	CTX-M-9 group	5 (1.0)	2 (1.5)	1 (1.4)
	cAmpC	8 (1.7)	5 (3.8)	2 (2.8)
*E*. *cancerogenus*	cAmpC	1 (0.2)	-	-
*E*. *aerogenes*	CTX-M-9 group	-	1 (0.8)	-
*Citrobacter spp*.	CTX-M-1 group	1 (0.2)	2 (1.5)	-
	CTX-M-2 group	-	1 (0.8)	-
	cAmpC	4 (0.8)	2 (1.5)	-
*S*. *liquefaciens*	CTX-M-1 group	2 (0.4)	-	-
*P*. *mirabilis*	CTX-M-9 group	-	1 (0.8)	-
*R*. *ornithinolytica*	CTX-M-9 group	-	1 (0.8)	-
*P*. *agglomerans*	CTX-M-9 group	1 (0.2)	-	-
**Nonfermenters**				
*A*. *baumannii*	OXA-58 + OXA-51	1 (0.2)	1 (0.8)	2 (2.8)
	OXA-51	2 (0.4)	4 (3.0)	-
*P*. *aeruginosa*	VIM-2	-	-	6 (8.5)

cAmpC, chromosomal AmpC β-lactamase (phenotypic result, only taken into consideration in Enterobacteriaceae)

In both centers on admission most patients were colonized with CTX-M-producing *E*. *coli* (NTORC: 38%; BTC: 53.8%; *p* = 0.01), followed by CTX-M-carrying *K*. *pneumoniae* (NTORC: 2.1%; BTC: 24.6%; *p*<0.001) and CTX-M-carrying *E*. *cloacae* (NTORC: 1.5%; BTC: 4.6%; *p* = 0.09). On day 14 CTX-M group-producing *E*. *coli* remained the most abundant coloniser (admission: 28.5%; day 14: 53.1%; *p*<0.001). Compared to admission the carriage rate on day 14 of ESBL-producing *K*. *pneumoniae* and *E*. *cloacae* rose from 1.3% to 9.5% (*p* = 0.001) and from 0.6% to 7.6% (*p* = 0.001) respectively. In all three groups carriage with CTX-M-9 group-producing *E*. *coli* was more common than with CTX-M-1 group-producing *E*. *coli*. Interestingly, multiple patients carried two bacteria of the same species (especially *E*. *coli*) with different CTX-M groups. Six *E*. *coli* isolates encoded ESBLs from both CTX-M-1 and CTX-M-9 groups, two CTX-M-9 group-producing *E*. *coli* isolates co-expressed the plasmid-mediated AmpC β-lactamase CMY-2. In all three groups, plasmid-mediated *ampC* resistance genes, *bla*_CTX-M-2_ group and *bla*_CTX-M-8/-25_ group genes were only rarely detected. No carbapenemase genes were detected in Enterobacteriaceae. OXA-carbapenemases were detected in a total of ten *A*. *baumannii* isolates, all of which carried the constitutive *bla*_OXA-51_ gene and four additionally carried the acquired *bla*_OXA-58_ gene. In the six *bla*_OXA-51_-only isolates an upstream IS*Aba1* was not detected. VIM-2 metallo-beta-lactamase was found in six isolates of *P*. *aeruginosa* (Table 3).

### Antibiotic susceptibility testing

Of the 666 Enterobacteriaceae isolates all except one (CTX-M-producing *P*. *agglomerans*) displayed non-susceptibility to cefotaxime and 55.5% non-susceptibility to ceftazidime. The highest rate of resistance among Enterobacteriaceae for non-β-lactam antibiotics was observed for trimethoprim-sulfamethoxazole (82%), gentamicin (51.4%) and ciprofloxacin (38.7%). Carbapenem-resistance in Enterobacteriaceae was very low, six isolates of *K*. *pneumoniae* and two isolate of *E*. *cloacae* were resistant to ertapenem. This was likely due to a combination of ESBL/AmpC β-lactamase and porin loss as phenotypic and molecular results were negative for carbapenemases. All six *bla*_OXA-51_-only *A*. *baumannii* isolates displayed intermediate susceptibility to meropenem and susceptibility to imipenem. The other four *bla*_OXA-58_-carrying *A*. *baumannii* and the *bla*_VIM-2_-carrying *P*. *aeruginosa* isolates were resistant to imipenem and meropenem and showed co-resistance to all the other antibiotic groups tested except for colistin. The results of antimicrobial susceptibility testing are shown in Table 4. Confirmatory phenotypic testing did not reveal antimicrobial resistance, possibly mediated by a β-lactamase gene not included in the PCR panel.

**Table 4:** Resistance rates of relevant Gram-negative multidrug-resistant bacteria.

Antibiotics	CTX-M-positive *E*. *coli %* (n = 536)	CTX-M-positive *K*. *pneumoniae %* (n = 55)	CTX-M-positive *E*. *cloacae %* (n = 31)	OXA-58-positive *A*. *baumannii %* (n = 4)	VIM-2 positive *P*. *aeruginosa %* (n = 6)
Cefotaxime	100	100	100	-	-
Ceftazidime	31.2	49.1	80.6	-	100
Meropenem	0	0	0	100	100
Imipenem	0	0	0	100	100
Ertapenem	0	10.9	6,5	-	-
Gentamicin	45.9	92.7	74.2	100	100
Tobramycin	14.6	70.9	74.2	100	100
Ciprofloxacin	38.1	54.5	45.1	100	100
Trimethoprim-sulfamethoxazole	80.8	96.4	96.8	100	-
Nitrofurantoin	1.9	-	-	-	-
Tigecycline	0	-	-	-	-
Chloramphenicol	20.5	61.8	83.9	-	-
Colistin*	ND	ND	ND	0	0

-, no clinical breakpoint available for disk diffusion method; ND, not determined;

*determined by gradient test

### Integron sequencing and genotyping of carbapenemase-producing isolates

The *bla*_VIM-2_ gene in *P*. *aeruginosa* was present on a class 1 integron with a 3’-conserved sequence consisting of a *tniC* gene. The integron array was composed additionally of the two aminoglycoside resistance genes *aacA7* and *aacC5b* and the dihydrofolate reductase gene *dfrB5* (gene cassette array In559 according to the INTEGRALL database). The *bla*_VIM-2_ gene was located on the chromosome. PFGE genotyping of the six *P*. *aeruginosa* isolates revealed four isolates that were genetically related to each other and the remaining two were classified as singletons. All *bla*_VIM-2_-carrying *P*. *aeruginosa* isolates were confirmed to be sequence type 235. All four *bla*_OXA-58_-carrying *A*. *baumannii* isolates were confirmed to be sequence type 642.

## Discussion

The main finding in this study is a high colonization rate of Gram-negative multidrug-resistant organisms (GN-MDRO) producing β-lactamases in the study population. In Mongolia, there is little data available regarding antimicrobial resistance and to our knowledge this is the first surveillance study that estimates faecal carriage of GN-MDRO. The high carriage rate of GN-MDRO on admission (42.4% in the trauma hospital and 69.2% in the burn hospital) is mainly due to dissemination of CTX-M-producing *E*. *coli* with more than one third of all patients admitted to the trauma hospital and every second patient to the burn hospital being colonized. ESBL-producing *Klebsiella* spp., *Enterobacter* spp. and other Enterobacteriaceae were found at a much lower rate; unsurprisingly as in the community setting ESBL-producing *E*. *coli* is known to play a bigger role, both as a normal intestinal commensal in humans and a major pathogen [2]. *E*. *coli* and *K*. *pneumoniae*, the main species identified, are worldwide the predominant organisms harbouring ESBL genes, the CTX-M enzymes representing the major mechanism of 3^rd^ generation cephalosporin resistance [2]. In all Enterobacteriaceae 3^rd^ generation cephalosporin resistance was nearly exclusively mediated by CTX-M-9 group and (to a lesser extent) by CTX-M-1 group enzymes. Two Mongolian studies have reported the presence of CTX-M enzymes in *E*. *coli* and *Klebsiella* isolates in clinical samples [10, 11]. And a similar spread of theses specific resistance gene groups were reported from China in *E*. *coli*, where the CTX-M-9 group was also the most abundant type [29].

On a global scale the carriage rate of ESBL-producing Enterobacteriacae in the community differs greatly ranging from low-prevalence countries like Northern-Europe to high prevalence regions like South-East Asia [2]. The observed high carriage rate at admission in this study is comparable to data from neighbouring China, where an alarming high prevalence of MDRO has been described, e.g. a 42% carriage rate of ESBL-producing Enterobacteriaceae in healthy individuals [5]. Interestingly in the bordering province Inner Mongolia 57% of community-acquired *E*. *coli* infections were ESBL producers [29]. The main reservoirs of the different Gram-negative species are the environment (city and hospital) and the gut of animals and humans, which explains the possible routes of acquisition [2]. A major risk factor for resistance development is the unregulated antibiotic consumption. Antimicrobials, despite the existing laws in Mongolia, are commonly purchased without prescription [13]. We also collected data about recent antibiotic therapy from a subgroup of 631 patients at admission to the trauma centre. As the data collection was anonymous no correlations can be made to the GN-MDRO-carrier status. Nevertheless 50% had taken antibiotics within the six months prior to admission (data not shown).

Our study also highlights the acquisition of GN-MDRO within the hospital setting. In the patients of whom swabs were taken after two weeks a significant number of patients (62.7%) were colonized with a new GN-MDRO and carried more than one GN-MDRO. The carriage rate of typically hospital-acquired multidrug-resistant species like *Klebsiella* spp. was more pronounced in the hospital setting. This can possibly be explained by nosocomial transmission, probably due to a less rigorous infection control practice in the hospital. Little is known about hospital-associated infection rates in Mongolia and resistance in clinical isolates since a surveillance system is not well established [8]. On the other hand, pre-hospital (asymptomatic) colonization is often only revealed once selection pressure is exerted in the hospital setting by prescription of antibiotics, so the high number might not reflect the true intrahospital transmission rate. The predominant role of ESBL-producing *E*. *coli* may support this hypothesis.

From the clinical point of view, this data of a high ESBL carrier rate is alarming as many critically ill patients in Mongolia are treated initially with 3^rd^ generation cephalosporins. For severe ESBL-driven infections, carbapenems are the drugs of choice. Unfortunately, carbapenems are not widely available in Mongolia [7]. We were able to show relevant co-resistances for Enterobacteriaceae against frequently used antibiotics such as trimethoprim-sulfamethoxazole, gentamicin or ciprofloxacin in our isolates, further obstacles for a therapy. This phenomenon could be explained by the widespread use or abuse of these antimicrobials, but also by the presence of the resistance genes on common integrons and plasmids [3].

Carbapenemase-producing organisms, only rarely detected in our study population, are certainly the most worrisome in the emergence of antimicrobial resistance and the prevalence is growing in Asia [4]. Carbapenemases were detected in both hospitals in four *A*. *baumannii* isolates (ST 642) driven by OXA-58 enzymes. The same sequence type was already described in clinical specimens in another hospital in Ulaanbaatar (First Central hospital) in 2013 [9]. We were also able to detect the metallo-β-lactamase VIM-2 in six carbapenem-resistant *P*. *aeruginosa* isolates (ST235). This is to our knowledge the first report of its presence in Mongolia. The unusual structure of the VIM-2 integron (In559) ending with a *tniC* gene shows the variability of these mobile genetic platforms and it was previously also reported from neighbouring country Russia with a near endemic presence of VIM-2-carrying *P*. *aeruginosa* of the same sequence type (ST235) throughout the country [27]. Both countries, Mongolia and Russia, have strong historical connections. Another important clinical fact of the discovery of VIM-2-producing *P*. *aeruginosa* is that they were found in the burn hospital. Especially burn patients are at risk of acquiring nonfermenters such as *P*. *aeruginosa* [30]. Despite being detected on admission to the burn hospital theses bacteria are a probable indicator for nosocomial transmission, as we did not find any MBL-carrying *P*. *aeruginosa* in the second hospital (NTORC) and four of six isolates were closely related (by PFGE) to each other.

The two study sites, a trauma and a burn hospital, not only differed significantly in their carrier rate (42.4% and 69.2% resp.), but also in gender and age. Gender and age can be explained by the different specialisation of the two hospitals. Men are more inclined to trauma and injuries as they pursue riskier activities at the workplace and at home (e.g. [31, 32]), whereas burns are a leading cause of injuries in children in Mongolia [33, 34]. In the BTC we did observe a colonization rate exceeding 50% in all age groups though significantly less in children. Other studies performed in the community in China and Germany did not observe any major age related differences [5, 35]. Also the significant higher colonization rate in the burn hospital with significantly more patients being colonized with more than one GN-MDRO may be due to a different study population. This may be due to previous hospitalisations in the burn hospital. Burn patients are prone to be (chronically) colonized by diverse Gram-negative species in the hospital setting, especially *P*. *aeruginosa* [30]. Alternatively swabs were taken in an undetected outbreak situation, in which patients were colonized within the three-day period of admission-sampling. Further studies including more patients included are needed to clarify this aspect.

There are a few limitations of this study. Firstly, colonization with a GN-MDRO may have occurred before the collection of the first swab (up to the third day of admission, approximately 48 hours, which is a defined interval for the definition of hospital-acquired infections [36]). Taking a swab directly at admission or even in out-patients might me more suited, but was not feasible in this study. Secondly the number of patients from whom a second swab was obtained was low, as most patients were discharged within two weeks and thus there might be a bias towards more critically-ill patients as they tend to stay longer in the hospital. To allow a clear analysis of in-hospital transmission data concerning antimicrobial resistance in clinical isolates from sites of infection is required. In addition, more surveillance data from the community is required, as our data indicate a high colonization rate at admission, but are possibly not representative for the Mongolian population, as we only performed this study at two very specialized centres.

## Conclusion

In summary, antimicrobial resistance in Gram-negative bacteria mediated by the extended-spectrum β-lactamases (CTX-M) represents a public health concern in Mongolia affecting the hospitalized patients and the community setting. This study emphasizes the importance of routine surveillance, appropriate infection control practice and antibiotic prescription practice to prevent further spread of Gram-negative multidrug-resistant organisms, especially of carbapenemases. Further studies exploring faecal carriage in healthy individuals and hospital-associated infections as well as antibiotic prescribing policies are needed.

## Supporting Information

S1 File Dataset of the patient characteristics and colonization status. (XLS)
